# Service evaluation of ‘GP at Door’ of accident and emergency services in Eastern England

**DOI:** 10.1017/S1463423624000707

**Published:** 2025-01-10

**Authors:** Julii Brainard, Aiden Rice, Gareth Hughes, Paul Everden

**Affiliations:** 1 Norwich Medical School, University of East Anglia, Norwich, UK; 2 North Norfolk Primary Care, Alkmaar House, Alkmaar Way, Norwich, UK

**Keywords:** emergency department, low acuity presentations, service evaluation, wait times

## Abstract

**Aim:**

We describe activity, outcomes, and benefits after streaming low urgency attenders to **G**eneral practice services at **D**oor of **A**ccident and **E**mergency departments (GDAE).

**Background:**

Many attendances to A&Es are for non-urgent health problems that could be better met by primary care rather than urgent care clinicians. It is valuable to monitor service activity, outcomes, service user demographics, and potential benefits when primary care is co-located with A&E departments.

**Methods:**

As a service evaluation, we describe and analyse GDAE users, reasons for presentation, wait times, outcomes, and co-located A&E wait times at two hospitals in eastern England. Distributions of outcomes, wait times, reasons for attendance, deprivation, and age groups were compared for GDAE and usual A&E attenders at each site using Pearson chi-square tests and accelerated time failure modelling. Performance in a four-hour key performance indicator was descriptively compared for co-located and similar emergency departments.

**Findings:**

Each GDAE saw about 1025 patients per month. Wait times for usual accident and emergency (A&E) care are relatively short at only one site. Reattendances were common (about 11% of unique patients), 75% of GDAE attenders were seen within 1 hour of arrival, 7% of patients initially allocated to GDAE were referred back to A&E for further investigations, and 59% of GDAE patients were treated and discharged with no further treatment or referral required. Pain, injury, infection, or feeling generally unwell each comprised > 10% of primary reasons for attendance. At James Paget University Hospital, 4.3%, and at Queen Elizabeth Hospital, 16.1% of GDAE attendances led to referral to specialist health services. GDAE attenders were younger and more socially deprived than attenders to co-located A&Es. Patients were seen quickly at both GDAE sites, but there were differences in counts of specialist referrals and wait times. Process evaluation could illuminate reasons for differences between study sites.

## Introduction

Attendances at UK National Health Service (NHS) accident and emergency departments (A&Es) have risen steadily since the 1990s, for diverse and many reasons (Kmietowicz, 2018). In 2004, NHS A&Es were set a target (key performance indicator, KPI) to admit, transfer for treatment, or discharge at least 95% of attending patients within four hours of presentation (4hKPI; Mortimore and Cooper, 2007). Waits above 5 hours in A&E have been linked to higher mortality within 30 days after attendance (Jones *et al.*, 2022) as well as longer hospital stays for patients admitted from A&E (Osborne, 2023). Most NHS A&Es have failed to meet the 4hKPI target in most time periods, since 2016 (Appleby, 2019). There is thus huge ongoing interest in innovative ways to better manage the needs of persons attending A&E to try to help A&Es achieve fast care delivery and reduce risk of health harms associated with delays to treatment or inappropriate treatment.

Previous observations (O’Keeffe *et al.*, 2018; Morris *et al.*, 2018; Ismail *et al.*, 2013; National Guideline Centre, 2018) suggested that 15%–44% of emergency department attenders in Britain sought health care for conditions that are suitable for other health services, especially primary care, where care could be administered by general practitioners (GPs) and/or advanced nurse practitioners (ANPs). Putting A&E attenders in need of GP-level treatment on a more appropriate pathway seems likely to produce many benefits: fewer breaches of the 4hKPI, faster resolution of health complaints for all, more proportionate investigation and treatment pathway, and better chances of continuity of care (Ismail *et al.*, 2013). During the COVID-19 pandemic, shorter waiting times and faster treatment were also desirable to reduce risk of nosocomial COVID-19 transmission (Chu *et al.*, 2020).

Since 2011, it has been increasingly common for GPs to be incorporated into A&E streaming and for GP services to be distinctive service areas within or adjacent to NHS A&Es (Uthman *et al.*, 2018). NHS five-year planning (2017) stated that every A&E should put in place ‘front-door clinical streaming’, to quickly find the most appropriate pathways for A&E attenders. One way to implement such streaming is via an immediately available GP-level service as alternative to A&E. Here we evaluate programmes that offer the full range of usual GP services to walk-in patients at two acute care providers in England. In the context of a service evaluation study design (Twycross and Shorten, 2014), using data routinely collected by the service providers and published data about co-located A&Es, we aimed to describe aspects of service provision including activity (counts of presentations), reasons for presentation, socio-demographic profiles of service users, any differences in waiting times, frequency of movement to a more urgent pathway, service user satisfaction, data about reattendances, and concurrent 4hKPI performance in co-located A&E compared to similar A&Es elsewhere in England.

## Methods

### Service description

GP at door of A&E (GDAE) services were commissioned by the Norfolk and Waveney integrated care system (N&WICS), in coastal eastern England. Most (97% of) hospital attendances by the approximately one million patients registered with N&WICS are at three acute care providers (Brainard *et al.*, 2022). A pilot GDAE service ran at the region’s largest acute care centre (Norwich and Norfolk University Hospital, NNUH) from December 2019 to February 2020 and is described elsewhere (Aldus *et al.*, 2022). The NNUH evaluation found that the GDAE model was feasible, acceptable to patients and clinicians, and was concurrent with apparent reduced demand for co-located A&E services. The GDAE service hoped to provide patient and system benefits: converting unplanned to planned treatment; providing clinician access to full primary care records, which were used in assessment; primary care records to be updated immediately; reduction in unnecessary investigations; and more appropriate risk management.

GDAE services were initiated and replicated in the NNUH format at the other two secondary care providers in N&WICS in late 2021/early 2022: Queen Elizabeth Hospital (QEH) and James Paget University Hospital (JPUH). The GDAEs at JPUH and QEH ran 7 days/week, 9am–9pm. Clinical GDAE staff typically comprised one GP and one ANP. Data about the GDAEs were available from service initiation, 16 months at the JPUH (October 2021–January 2023) and 12 months at the QEH (Feb 2022–January 2023).

The GDAE care pathway is illustrated and described at length elsewhere (Aldus *et al.*, 2022). In brief, patients attending the A&E walk-in entrance (arrival not by ambulance) were initially triaged by a GP or ANP to attend GDAE service or usual A&E care. Bespoke triage criteria were applied according to criteria set out in the Supplementary Material List S1 and clinical acumen. Patient care during GDAE attendance was meant to be identical to care available at patient’s usual NHS-registered GP surgery. GDAE patients were booked in and asked for consent to access their primary care records. Care was provided by a GP or ANP and primary care records were updated by these same staff or other supporting administrative staff (none of whom were otherwise involved in service evaluation). Transfer to the usual A&E pathway was possible at any time.

### Activity and outcomes

Ethical approval for this study using these data was granted by the University of East Anglia Faculty of Medicine and Health Ethics Committee (Reference: ETH2122-1954, 22 June 2022). Written consent from individual patients was not required because this is a service evaluation that used fully anonymous administrative data. The GDAE services were described and evaluated using the data and outcomes listed below, from routinely collected administrative data.

– Service user descriptions (sex, age, and deprivation levels)

– Total GDAE activity (attendances, appointments, and outcomes from appointments)

– Wait times

– Outcome after completing GDAE appointment

– Frequency of reattendance

– Most common reasons for attendance to GDAE, including among persons who reattended during the monitoring period

– How well each of the co-located A&Es concurrently met 4hKPI compared to historical data or similar A&Es

### Data: service users

Data were routinely collected in electronic medical records (SystmOne and Symphony). The data described individual GDAE attenders who were residents in N&W or out of area, using the below fields:

– Unique patient identifier
– GDAE location
– Date of attendance
– Time at booking in
– Appointment status (eg., finished or cancelled)
– Gender
– Age (in whole single years)
– Deprivation group decile and quintile
– Reason for attending
– Treatment outcome (eg., referral, discharge or not recorded)
– Wait from arrival time to time seen (minutes)

The Index of Multiple Deprivation 2019 (McLennan *et al.*, 2019) is a national ranking of relative deprivation in residential areas. These ranks were available in deciles and quintiles (quintiles are 1 = most deprived to 5 = least deprived) relative to all England. Available (in system coding at time of booking) reasons for attendance (descriptions of primary complaint when patient presented) are listed in Box 1.

Box 1. Primary reasons for attending A&E, as recorded at GDAE booking N&W A&Es.

**Table:** 

Unwell/Cough/Headache/Fever	Gynaecological
Swelling/Lumps/Bumps	Genitourinary symptoms
Septicaemia	Gastrointestinal problem
Seizure	Eye/Vision problem
Rash/Skin/Cyst/Allergy/Discolouring	Eating/Drinking problem
Pregnancy	Ear/Hearing problem
Poisoning	DVT
Pain/Soreness	Dressings/PostOp problems
Other general symptoms	Dizziness/Faint/Confusion
Nervous system symptoms	Diabetes
Nausea and vomiting	Dental
Medication	Burn/Scald
Insect bite	Bleeding
Injury/Fall	Asthma/breathing problem
Infection/Ulceration	Anxiety/Shuddering/Mental Health
Hernia	Animal bite
Heart/Circulatory

### Data: four-hour target for discharge or admission for NHS A&Es

NHS England publishes statistics (percentages) for how many patients completed their A&E visit within four hours. Just prior to GDAE initiation, in September 2021, the JPUH A&E had 7135 completed attendances, and the QEH had 6487 attendances. From NHS England datasets, we extracted 4hKPI values for prior 12 and subsequent months after GDAE services started at JPUH and QEH, as well as for all (n = 23) comparator type 01 (full range of urgent care services provided) NHS emergency departments that had somewhat similar total attendances (between 4000 and 10,000) in September 2021. The 23 comparator sites are listed in Supplementary Material Table 1. Many initiatives to try to improve the 4hKPI were concurrently happening widely in the NHS, although we did not know their full extent at each of these specific 23 comparator sites. Rather, our rationale was to try to explore whether the 4hKPI at JPUH and QEH had improved, deteriorated, or remained static compared to KPI performance over a similar period at comparable NHS A&Es. We also descriptively compared the 4hKPI at JPUH/QEH, pre- and post-implementation of their GDAE services.

### Service user satisfaction

We summarize responses to an online survey that service users were invited to take; survey questions are in Supplementary Material List 2. These questions were bespoke and designed by service managers without validation or piloting prior to data collection. The survey includes one question that is very common in consumer satisfaction surveys in the UK ‘Would you recommend our Service to your friends and family?’.

### Analysis

Evaluation of service outcomes was possible with only normally collected service data. Therefore, analysis of the service was pragmatic and designed to maximize information from data that were collected to support service monitoring. We focus results in this article on outcomes related to concurrent operational priorities for NHS commissioners, which included a nationally important performance indicator for the co-located emergency departments (4hKPI), reattendance rates (which has a bearing on clinical safety because reattendance may indicate failed initial care), and demographic associations with service demand and patient satisfaction. Our approach was to apply descriptive statistical analysis, including comparisons between the JPUH and QEH using Pearson chi-square test of proportionality difference, Mann–Whitney U test, and survival analysis. Statistical calculations were undertaken in RStudio v. 4.4.0. The significance threshold was set at p < 0.05.

Counts of patients treated by each routine (not GDAE) A&E service (QEH or JPUH) were supplied by N&WICS for 2021 and 2022 calendar years, broken down into the ten nationally ranked deprivation deciles and disaggregated into nine age bands with boundaries (in years): 0-4, 5-14, 15-24, 25-34, 35-44, 45-54, 55-64, 65-74, 75+. To determine if GDAE users were different from usual A&E users with respect to age or deprivation, the distributions of counts of patients in deprivation deciles and the preceding age group categories were compared using Chi-square tests for individual A&Es in 2021 and 2022, and between individual A&Es with patients at each co-located GDAE site in 2022. Note that a unique patient’s visit could be included in GDAE and also included in the concurrent A&E statistics if that patient was transferred from the GDAE to A&E. However, such transfers were rare, as documented below. We narratively describe reasons for presentation including reattendance. We do not apply a statistical test to compare proportions of reasons attributed to single and repeat attenders. Because of the large number of categories and small counts in some categories, statistical testing for differences was unlikely to yield informative results.

Kaplan–Meier survival curves (Schober and Vetter, 2021) were generated to visually compare differences in the wait times between the two GDAE sites from booking in time until patient was seen (when GDAE care appointment started). A variety of non-parametric distributions (Supplementary Material Figure S1) were evident in the wait times and in candidate co-variates: age, deprivation, and distance from registered home address. Testing for significant differences between wait times at the two sites unadjusted by other factors was done in two ways: using Mann–Witney U test (comparison of medians) and log-rank sum test (Schober and Vetter, 2021) between the Kaplan–Meier curves for the two sites. We then modelled adjusted associations using accelerated failure time (AFT) models with key candidate co-variates: sex, age, deprivation decile, or distance from home address. The condition of proportional hazards was not met which would have enabled Cox proportional hazards regression (Orbe *et al.*, 2002; DiSaia *et al.*, 2017) but AFT was an appropriate alternative survival model. The results of the log-rank test are valid but its power was reduced because the assumption of proportional hazards was not met with these data (Rizopoulos, 2023). Although this power reduction was somewhat mitigated by the very large sample size, the log-rank test also cannot reflect differences identified in multivariable models, which also made generating a multivariable AFT model informative.

Most available distributions for AFT models in available software packages cannot handle zero values, which were present in our dependent variable (many wait times were zero minutes); we, therefore, added one (1 minute) to the dependent variable (wait from arrival to being seen) for all records with a recorded time. We felt this adjustment was acceptable because the addition of one minute was negligible to the clinical outcome (speed of being seen). The preferred AFT model was constructed in stepwise fashion starting with all candidate co-variates and eliminating them singly until only those with *P* < 0.05 remained. The AFT model followed a Weibull distribution. We confirmed that this had reasonable fit in final model by plotting Kaplan–Meier residuals (Rizopoulos, 2023).

Concurrent statistics for the 4hKPI are provided and discussed narratively before and after the N&W GDAE services were deployed (first 12 months only), comparing JPUH and QEH with their own historical data and similar A&E departments in England. We describe the differences narratively by comparing relative rank of the study site A&Es within this group of A&ES, before and after the GDAE services became operational, to see if there were relative concurrent improvements in the 4hKPI at the GDAE sites.

## Results

### Service overview: activity, users and their outcomes

Supplementary Material Figure S2 shows the counts of records received and used at different points in the analysis. Table 1 in main text shows overview information for each and both services. Over 99% of patients booked into GDAE attended their appointments. Other bookings did not proceed due to cancellation (by user or service) or when patient did not attend. Detailed demographics and outcomes are provided only for patients who completed appointments; denominators for percentage calculations exclude records where an attribute was not recorded. Median age and age distribution of attenders were nearly identical at the sites (median = 33 years, range 0–100). Items in bold font in Table 1 had significant proportional differences (chi-square test) between JPUH and QEH. JPUH patients were significantly more likely to come from the most deprived quintile areas, 36.9% vs. 26.1% at QEH. QEH had more attendances by patients resident outside Norfolk and Waveney. Of unique persons who attended each GDAE, there were repeat attendances by 1944 persons at JPUH (12.6% of unique 15,398 service users) and 867 at QEH (9.1% of 9533 GDAE service users). Among completed appointments, repeat GDAE service users accounted for 18.4% of GDAE attendances (1959/10629) at QEH and 25.6% of attendances at JPUH (4634/18087). The most GDAE attendance by an individual at each site was 14. The proportions of several treatment outcomes differed significantly between sites in some respects: QEH patients were more likely to be referred to other services (A&E or specialisms) or to be referred back to their GP with recommendations for further treatment/investigations.

**Table 1.** **G**eneral practice services at **D**oor of **A**ccident and **E**mergency departments **Service activity and users** **overview**

	JPUH: n, %	QEH: n, %	All: n, %
Appointments made	18,212	10,655	28,867
Appointments attended	18,087, 99.3%	10,626, 99.7%	28,713, 99.5%
*Of 28,713 who completed an appointment with GDAE …*			
Females	9276, 51.2%	5578, 52.5%	14,854, 51.7%
Median Age (IQR)	33, 14–55	32, 12–55	33, 13–55
In most deprived quintile	**6658, 36.9%**	**2751, 26.1%**	9409, 32.8%
Resident in N&W	**16,763, 92.2%**	**7682, 72.3%**	24,445, 85.1%
*Reattendance*			
Unique patients	15,398	9533	24,931
# Patients who made 2 visits	**1484, 9.6%**	**726, 7.6%**	2210, 8.9%
… 3 visits	**311, 2.0%**	**93, 0.98%**	404, 1.6%
…. 4 visits or more	**149, 0.97%**	**48, 0.50%**	197, 0.79%
***Outcome after being seen***			
*Discharged after…*			
Treated with no further treatment required	**11,629, 64.4%**	**5220, 49.2%**	16,849, 58.8%
Not treated with no further treatment required	2618, 14.5%	1468, 13.8%	4086, 14.3%
Unclear if treated, but no referral or further treatment required	583, 3.2%	303, 2.9%	888, 3.1%
*Referred to…*			
GP, further action recommended	**1423, 7.9%**	**904, 8.5%**	2327, 8.1%
GP, for watchful waiting	21, 0.1%	1, 0.01%	22, 0.1%
Specialty service	**770, 4.3%**	**1706, 16.1%**	2476, 8.6%
A&E immediately	**1007, 5.6%**	**1007, 9.5%**	2014, 7.0%
Patient declined further treatment or referral	3, 0.02%	(none)	3, 0.01%

*Note*: Outcomes were mutually exclusive. If patients were treated prior to a referral was not recorded. Items in bold were significantly different between JPUH and QEH at p < 0.05.

### Demographic comparison of service users at A&E and GDAE: deprivation and age profile

Table 2 shows distributions (percentages of all service users) in each deprivation or age group, at each site and service, for 2021 and 2022. The data are reported in groups, top half is age breakdown, and bottom section is deprivation, while left side is JPUH and right side is QEH. The 2022 GDAE data are far right column within each of the four sub-groups. The deprivation and age distributions appear to be very similar at each main A&E site between 2021 and 2022. The distributions in 2022, however, appear somewhat younger and more deprived of GDAE services at each site compared to concurrent co-located usual A&E users. For instance, 15.1% of GDAE attenders to the QEH were in the most deprived decile, compared to 9.8% of attenders to usual QEH A&E in 2021 and 2022. That the deprivation differences were statistically different for GDAE services and the co-located A&Es in 2022 was confirmed with chi-square tests, which had p << 0.001 for proportional differences at both JPUH and QEH. Similarly, for age distributions in 2022, we confirmed that GDAE service users were significantly younger than routine A&E patients, by applying the chi-square test for these age groups in 2022, which resulted in *P* << 0.001 at both JPUH and QEH.

**Table 2.** Deprivation and age-distributions at study sites, 2021 and 2022

*Age distribution: JPUH*				*Age distribution: QEH*		
	**A&E**		**GDAE**	**A&E**		**GDAE**
**Years**	**2021**	**2022**	**2022**	**2021**	**2022**	**2022**
0 to 4	8.5%	8.6%	12.5%	8.1%	8.3%	14.6%
5 to 14	9.3%	10.9%	12.2%	7.9%	8.7%	12.7%
15 to 24	11.2%	11.1%	12.0%	10.8%	10.0%	12.1%
25 to 34	11.8%	10.9%	14.2%	12.2%	11.2%	14.4%
35 to 44	9.7%	9.2%	11.8%	9.6%	9.5%	11.1%
45 to 54	9.7%	9.1%	10.8%	10.0%	9.2%	9.7%
55-64	10.2%	10.0%	10.5%	10.4%	10.4%	9.7%
65 to 74	10.4%	10.2%	8.0%	10.7%	10.8%	7.8%
75+	19.3%	20.0%	7.9%	20.4%	21.8%	8.0%
Total attendances	76,888	76,088	14,273	72,505	74,847	10,394

*Deprivation distribution: JPUH*				*Deprivation distribution: QEH*		
	**A&E**		**GDAE**	**A&E**		**GDAE**
**Decile**	**2021**	**2022**	**2022**	**2021**	**2022**	**2022**
1	24.2%	23.5%	26.5%	9.8%	9.8%	15.1%
2	11.8%	11.5%	10.1%	11.5%	11.6%	10.8%
3	10.8%	10.7%	11.4%	18.2%	18.5%	16.2%
4	13.1%	13.7%	13.1%	17.4%	17.4%	20.6%
5	13.3%	13.2%	12.8%	19.0%	18.8%	14.6%
6	11.4%	11.5%	8.9%	12.7%	12.7%	9.5%
7	4.6%	4.6%	6.2%	4.7%	4.7%	5.6%
8	5.3%	5.6%	4.9%	1.8%	1.9%	3.1%
9	4.2%	4.3%	4.8%	3.1%	2.9%	3.2%
10	1.4%	1.3%	1.3%	1.8%	1.8%	1.2%

*Notes*: JPUH = James Paget University Hospital. QEH = Queen Elizabeth Hospital. A&E = accident and emergency. GDAE = GP at door of A&E.

### Reattendance and reasons for presentation

Primary reasons for presentations that comprised at least 1% of visits are listed individually in Table 3; all other reasons (such as ‘diabetes’) each comprised < 1% of visit reasons and are grouped under ‘Other conditions’. There were 867 persons who attended the QEH GDAE more than once in the 12 months of monitoring, and 1944 persons who attended the JPUH GDAE more than once in 16 months of service operation. Persons who attended more than once had a similar distribution of reasons as single-occasion attenders. The largest difference between one-time and repeat attenders is that reattendances at QEH were much more likely to be for feeling ‘generally unwell’: 18.3% among repeat attenders vs. 13.5% for full group.

**Table 3.** Primary reason for attendance to **G**eneral practice services at **D**oor of **A**ccident and **E**mergency departments services

	All attendances			Attendances by repeat attenders	
Primary reason for attendance (where recorded)	JPUH n = 17295	QEH n = 10605		JPUH n = 4446	QEH n = 1957
Animal bite	2.0%	*< 1 %*		1.5%	*< 1 %*
Asthma/Breathing	2.6%	2.1%		3.1%	2.3%
Dental	6.1%	4.6%		6.1%	4.4%
Dressings/PostOp problems	2.7%	1.2%		3.9%	2.3%
DVT	< 1%	1.5%		< 1 %	1.0%
Ear/hearing problem	3.4%	3.7%		4.8%	5.0%
Eye/Vision problem	4.0%	7.3%		3.1%	5.1%
Gastrointestinal problems	4.0%	5.5%		3.8%	6.3%
Gynaecological	1.1%	1.7%		1.3%	1.6%
Infection/Ulceration	11.4%	9.6%		13.3%	11.4%
Injury/Fall	13.3%	12.3%		8.2%	7.5%
Insect bite	1.3%	*< 1%*		1.1%	*< 1 %*
Nervous system symptoms	*< 1%*	1.2%		*< 1 %*	1.2%
Pain/Soreness	17.9%	16.9%		15.7%	14.0%
Rash/Skin/Cyst/Allergy/Discolouring	4.5%	4.1%		5.4%	6.5%
Swelling/Lumps/Bumps	1.2%	1.7%		1.2%	1.7%
Unwell/Cough/Headache/Fever	10.2%	13.5%		12.8%	18.3%
Other stated conditions (each <1%)	14.4%	13.1%		14.6%	11.3%

*Note*: Totals are for visits with presenting complaint recorded; there were additional visits without reason for presentation recorded. PostOp = postoperative problems.

### Timing of attendance and waits to be seen

**Figure 1.**
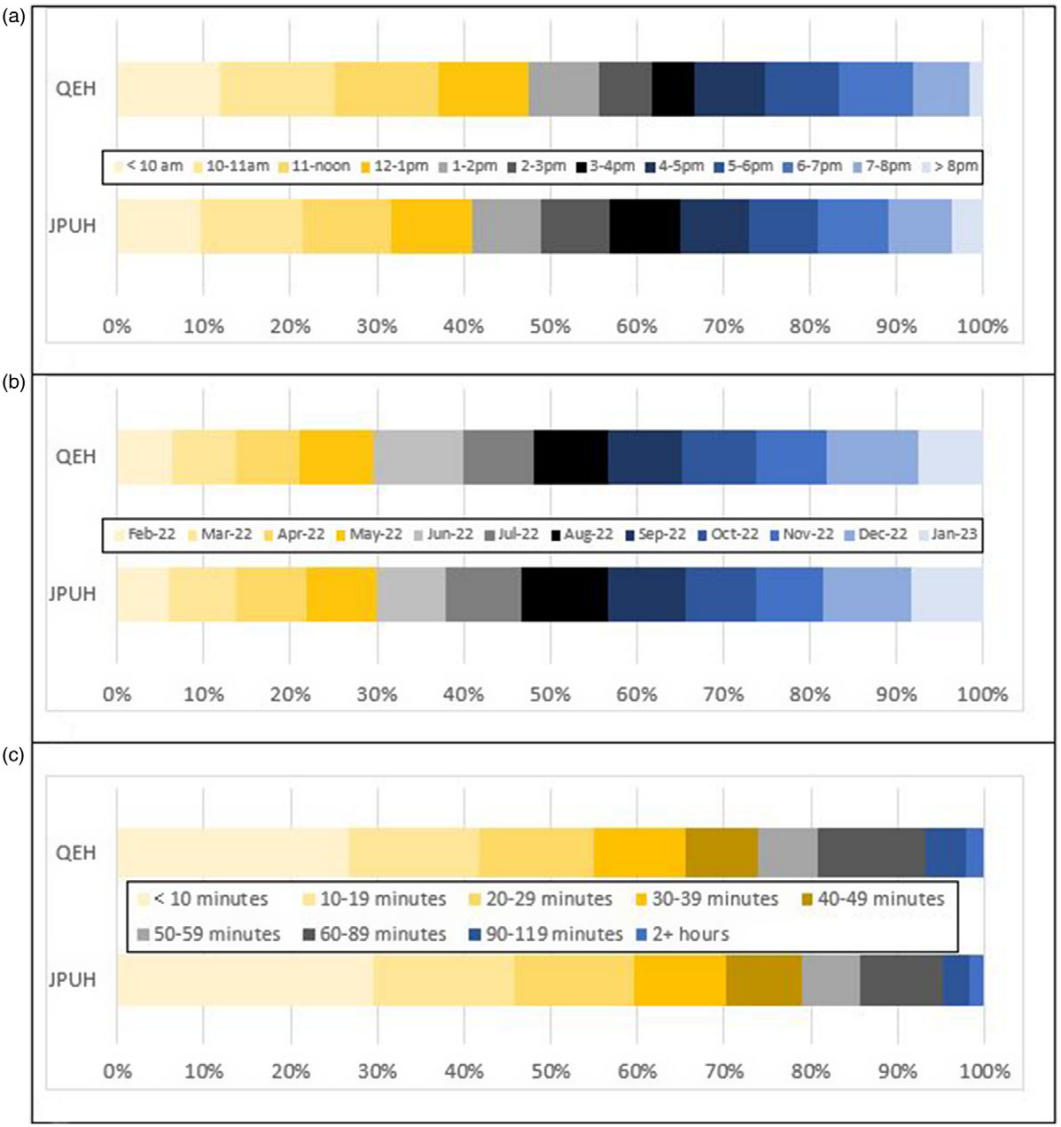
Service activity for completed **G**eneral practice services at **D**oor of **A**ccident and **E**mergency departments service users. *Notes*: a. time of day for arrivals. b. arrival proportions in most recent 12 months of service. c. elapsed time from arrival to being seen.

For patients who completed appointments, Figure 1a–1c depicts these aspects of service attendance: arrival time (resolved to nearest hour); month of attendance (last/concurrent 12 months only); and wait from arrival time to time seen (whole minutes). There was negligible difference between sites with respect to proportion of attendance in any specific calendar month (Figure 1b). However, both times of arrival (1a) and wait times (1c, from arrival to being seen) seemed different between sites. Most (55.6% of) QEH patients arrived by 2 pm, compared to 48.9% of JPUH patients. This difference was significant in a chi-square test, p << 0.001. Durations of actual appointments at both sites were similar: QEH median 15 (IQR 11–21) and JPUH median 16 (IQR 11–22). We also looked at proportions arriving by day of week (eg., Monday, Tuesday…) and found no significant difference between sites (data not shown).

Most patients (about 75%, Figure 1c.) were seen within one hour after arrival at either site. Wait-time differences between the two GDAE services were compared with survival analysis. Supplementary Figure S3 shows a Kaplan–Meier curve for 28,786 GDAE service wait times. Elapsed time from arrival to being seen was greater at QEH (*P* < 0.001, Mann–Witney U test), with QEH median elapsed time = 26 minutes (IQR 8–51), while the time from arrival to being seen at JPUH was median 22 minutes (IQR 7–45). A log-rank test for the differences was significant at *P* < 0.001 (chi-square test). However, in absolute terms, the difference between 26 and 22 minutes (n = 4) may be considered clinically negligible.

In AFT modelling with a Weibull distribution for wait-time differences between the two GDAE sites, age was a statistically significant predictor but not deprivation decile, patient gender, or distance from home address to the service location. This model is summarized in Table 4. The positive coefficient (e.g., for age) in the AFT model indicates longer wait times for higher values (i.e., older people had longer waits). JPUH is indicated as site 1 in this model, and QEH is site 2, so waits were found to be significantly longer (*P* < 0.001) at QEH. Kaplan–Meier estimates of the model residuals are plotted in Supplementary Material Figure S4 and visually suggest that assuming a Weibull distribution was acceptable.

**Table 4.** Accelerated Failure Time model for wait times from booking in to start of **G**eneral practice services at **D**oor of **A**ccident and **E**mergency departments appointment

Variables	Coefficient Value	95% confidence interval	p-value
Intercept	3.389	3.367, 3.412	< 0.001
QEH site	0.128	0.105, 0.153	< 0.001
Age	0.00149	0.00102, 0.0.00197	< 0.001
Log(scale)	0.00375	−0.00550, 0.0130	0.43

*Notes*: JPUH is reference site. Model using Weibull distribution, with chi-square = 148.56 on 2 degrees of freedom. 28,866 observations, number of Newton-Raphson Iterations = 7.

### Four-hour target statistics, study sites, and similar A&Es

**Figure 2.**
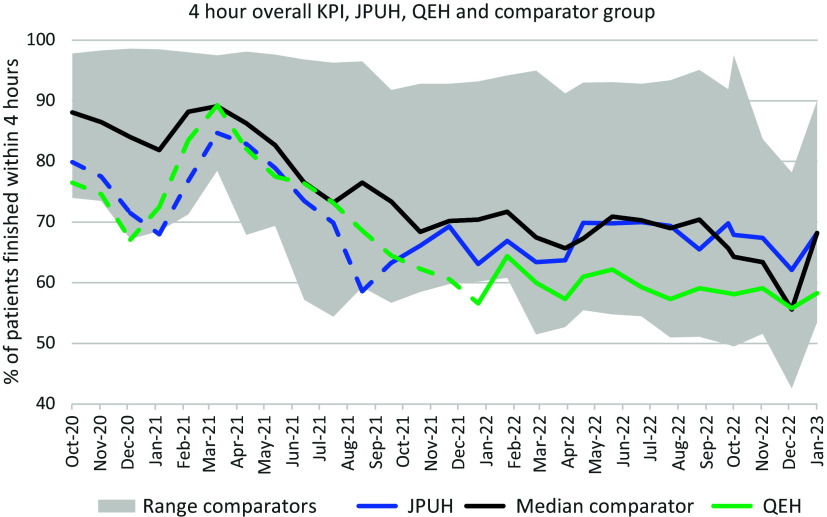
Four hours of presentation for all arrivals, James Paget University Hospital /Queen Elizabeth Hospital and comparator group of A&Es. *Notes*: Data source: National Health Service England. Comparator sites are defined in text (A&Es with similar attendance counts and facilities in September 2021). Dashed lines: before **G**eneral practice services at **D**oor of **A**ccident and **E**mergency departments (GDAE) service; solid lines: GDAE service in operation.

Figure 2 illustrates the overall 4hKPI performance at JPUH and QEH A&Es each month, compared to the group of similar size (activity level) A&Es in England that also provide a full range (Type 1) of emergency department services. In Figure 2, the 4hKPI of median performing comparator A&E is indicated with the central black line. The full range of comparator 4hKPIs is denoted by grey-shaded areas. Dashed lines indicate the period prior to the introduction of the GDAE services at JPUH/QEH (minimum 12 months before). The GDAE services did not coincide with consistently improved overall 4hKPI at JPUH or QEH compared to their own preceding 12 months of data. However, it makes more sense to consider the 4hKPI relative to the comparator group of A&Es, given that there is long-term deterioration in 4hKPI at all NHS A&Es, a decline which has accelerated since 2019 (Nuffield Trust, 2022).

Compared to similar A&Es, the JPUH and QEH 4hKPIs were consistently in the 50% of lower performing A&Es prior to GDAE introduction. After GDAE, the QEH usually remained among the 25% of lowest performing A&Es. Specifically, QEH had ranked 19 or greater in five of the 12 months February 2021 to January 2022, among these 25 A&Es. The QEH ranked 19 or greater in eight of the 12 months from February 2022 to January 2023, which suggests relatively worse performance after GDAE, and a tendency to be in the lowest quartile during these 12 months. The JPUH had ranked ≥ 19 in 8 of 12 months among this group of 25 in the 12 months prior to GDAE introduction (October 2020 to September 2021). However, in the first 12 months of GDAE operation (October 2021-September 2022), the JPUH 4hKPI rank in this group of 25 was no worse than 18 in any month and median JPUH rank was 13; there was strong apparent improvement in the JPUH 4hKPI relative to similar A&E services after the JPUH GDAE service was introduced.

### Service user satisfaction

Patients who gave data about satisfaction numbered 42 at JPUH and 32 at QEH (Table 5). There were many very positive comments about care received, naming individual clinicians, and specific points of gratitude. Most respondents said that there was nothing they could think of to improve the service. There were three negative statements in the open text responses: 1) one at-door streamer was ‘rude’; 2) excess walking distance from A&E entrance to entrance to GDAE; and 3) brusque manner of consultation GP. All negative comments were at the JPUH.

**Table 5.** Results of patient satisfaction survey

Question	JPUH	QEH	Combined
*Who referred you to A&E today?*			
No one/ brought self	61%	73%	65%
NHS111 (telephone/online advice service)	22%	6.7%	15%
GP advised attendance	9.8%	20%	14%
Chemist/pharmacist	2.4%	0%	1.4%
Nurse in A&E	2.4%	0%	1.4%
St. John Ambulance	2.4%	0%	1.4%
*Would you recommend the service to friends and family?*			
Yes	90%	97%	93%
No	10%	3%	6.9%
*Were you aware of this service prior to arrival?*			
Yes	15%	23%	18%
No	85%	77%	82%
*How did you find person who met you at the entrance …*			
Good or Excellent	86%	96%	90%
Fair	5%	3%	4%
Poor or Very Poor	2%	0%	1%
*Booking receptionist was …*			
Very helpful	93%	97%	94%
Somewhat helpful	5%	0%	2.8%
Neither helpful nor unhelpful	2%	0%	1.4%
Unhelpful or very unhelpful	0%	0%	0%
No receptionist only clinicians at arrival	0%	3%	1.4%
*How did you find consultation with clinician …*			
Good or Excellent	95%	100%	97.2%
Fair or Poor *(user entered both answers)*	2.4%	0%	1.4%
Poor	2.4%	0%	1.4%
Very Poor	0%	0%	0%
*How do you think we could improve the services?*			
No comment/Nothing needs improvement	76%	67%	72%
Wait times could improve	7.1%	6.7%	6.9%
Should be publicized more	2.4%	13%	6.9%
Other specific comments or suggestions	14%	13%	14%

## Discussion

There is a long-standing objective (NHS 2017) that every A&E should put in place ‘front-door clinical streaming’, to quickly find the most appropriate pathways for A&E attenders. One way to implement such streaming is with use of an immediately available GP-level service. Among about 28,000 arrivals who received GDAE care (Table 1 data), only about 2000 attendances were returned to the usual A&E pathway. Given the co-located A&E combined normally receive about 200,000 patients per year, a net reduction of 26,000 A&E patients in a 16-month period may seem significantly beneficial. Other recent analyses concluded that primary care clinicians working in A&Es did not increase efficiency of services, improve clinical outcomes, or patient or staff experiences (Scantlebury *et al.*, 2022; Gaughan *et al.*, 2022). Those studies noted that there is substantial variation in how primary care clinicians work at A&Es (Brant *et al.*, 2021), which may lead to inconsistent possible benefits. Wait times from arrival to being seen by the GDAE services were shown to be different between sites, and correlated with age and deprivation. The reasons for these findings and between-site differences probably require process evaluation (McGill *et al.*, 2020) to thoroughly understand.

The Norfolk and Waveney GDAE services coincided with (relative to each other and 23 other similar-sized A&Es) improvement in 4hKPI at JPUH and deterioration of the 4hKPI at QEH. We lack information about the diversity of wait-time reduction initiatives that we are sure were concurrently in operation at the 23 comparator sites. Potential reasons for differences between JPUH and QEH outcomes are easier for us to identify. For instance, lower inpatient bed capacity at the QEH may have delayed admissions and thus had the greatest impact on 4hKPIs. Concurrently during this period, the QEH had to contend with failing infrastructure, which may have undermined efficacy and quality of services (BBC News, 2022). Also relevant may be somewhat different aspects between the sites with respect to times of day when patients attend and their modes of arrival. Residents in more deprived areas (lower deciles) were over-represented among attenders to A&E and GDAE, while GDAE patients were much younger and more deprived than usual A&E patients, on average, at both sites. That A&E attenders with more minor problems tend to be relatively young is a finding in other recent research (O’Keeffe *et al.*, 2018). Our own findings likely also reflect arrival mode; in the UK, patients age 75+ are much more likely to travel to A&E in an ambulance (Lofthouse-Jones *et al.*, 2021), whereas the GDAE services were only offered to walk-in patients.

Reasons for attendance did not appear to be very different between sites or between single-occasion attenders versus reattenders. Clinical audits and process evaluation would also be helpful in understanding why QEH patients were more often referred to other services.

We hope to undertake future research to better understand barriers faced by N&W residents when trying to obtain GP-level care. We previously involved patient and public advisors to explore how A&E attenders with low acuity conditions may be asked about their experiences of seeking health care without conferring stigma. Collecting such data would provide specificity and focus on real-world barriers that people face and cause them to find it appealing to obtain unscheduled GP-level care by attending A&E. Preliminary comments from our public advisors include that from a patient’s perspective, A&E visits seem to provide more reassurance and faster resolution than visits to a GP surgery. GDAE format services may help to provide these benefits to persons who attend A&Es while reducing the risks of possible over-investigation and over-treatment (NHS Confederation, 2022), especially in an era when routine GP appointments seem very challenging to obtain (Hayward *et al.*, 2023).

### Strengths and limitations

To know whether the GDAE services described in this service evaluation have achieved the most appropriate level of care would require patient history audits by clinically qualified staff; we did not have resources to attempt that. We lacked access to sensitive individual patient clinical data to assess how often reattendances were for the same condition that caused initial presentation. We recommend to commissioners that evaluation of potential benefits or harms of GDAE format services should ideally include clinical audit to ascertain if reattendances tend to be for exacerbations of initial conditions or for unrelated health problems. Such audit results would, however, need to be interpreted with reference to frequency of reattendance to A&E itself for related conditions, and/or to community GP surgeries, for escalations of health problems that were recently treated by same type of service.

Performance of the 4hKPI at JPUH and QEH was only analysed descriptively; a large and different study design (randomized controlled trial) would be necessary to evaluate GDAE design services impacts on the 4hKPI robustly against other initiatives that try to improve the 4hKPI. We have not attempted cost-benefit analysis, which to be reliable, would be its own large separate exercise and many more sites to balance out unobserved mediating factors. We robustly looked for statistically significant differences in wait times between sites. This information helps to inform any future process evaluation, but we do not focus on wait-time outcomes in the main results because the typical wait-time difference was small while NHS commissioners are understandably more concerned with possible effects on the four-hour KPI for A&Es, demographic aspects of patient need, safety outcomes, demand generated for other services, and patient satisfaction. Our study benefited from a large and comprehensive dataset and careful collaboration between NHS administrators and research analysts to ensure that routinely collected data could be safely and anonymously analysed to ensure an informative service evaluation. ‘Lack of consistent evaluation’ is a chronic problem in the NHS (Roy, 2019), which means that commissioners struggle to know what benefits may have been achieved or what parts of process to focus on; our study attempts to redress this information deficit.

GDAE services in N&WICS appeared to avert more than 26,000 A&E attendances in a 16-month period. This may have helped cause a relative improvement in the 4hKPI at one of the study sites. The services have been busy and resulted in high satisfaction among service users. The JPUH service was somewhat more efficient than QEH (shorter wait times, fewer onward referrals) in spite of JPUH serving a more deprived (presumably with greater morbidities) population; this finding suggests there may be opportunities for efficiency gains at the QEH GDAE. Reattendance is common at both sites, which could be incidental or could arise from ineffective care at an earlier appointment or may reflect an undesirable outcome: some attenders may now prefer GDAE over their usual GP service. GDAE data could be used to help frequent attenders find more appropriate service pathways. Process evaluation would be useful to understand differences in GDAE outcomes at each site.

## Supporting information

Brainard et al. supplementary material Brainard et al. supplementary material

## Data Availability

Data are only available as reported in this manuscript in aggregate. Data for individual patients are not publicly available.

## Supplementary material

The supplementary material for this article can be found https://doi.org/10.1017/S1463423624000707
