# Supplementary material for: Service evaluation of ‘GP at Door’ of accident and emergency services in Eastern England
Source: Prim Health Care Res Dev. 2025 Jan 10;26:e5. doi: 10.1017/S1463423624000707 (PMC11735119; doi:10.1017/S1463423624000707)
Supplement: Brainard et al. supplementary material [file S1463423624000707sup001.docx]

# Supplementary Material

**List S1. Triage Inclusion/Exclusion criteria:**

The service does not have a strict inclusion/exclusion criterion. It relies on the clinical acumen of the clinicians working in the service. The criteria that are in place are outlined below:

Patient Inclusion Criteria

* All patients who self-present to the emergency department will have the opportunity to access a general practice appointment if they are registered with a Norwich, North Norfolk, West Norfolk, Great Yarmouth and Waveney, or South Norfolk GP Practice and are assessed by a clinician to be suitable for primary care.

* Patients out with this catchment area may also be seen by the service due to an update in the process and agreement. Details of their review by the service will be sent to their own GP surgery.

* Children may be seen by the service for minor illness complaints where appropriate as assessed by the GP or ANP.

Patient Exclusion Criteria

* Any patient requiring intervention or investigation within ED

* Non-traumatic chest pain

* Any patients with signs or symptoms of stroke

* Any patients, that are not registered with a GP Surgery within the UK

* All children presenting to ED with injuries

* Children under three months

**Table S1.** Study sites (yellow fill) and comparator sites (green fill) in England, UK. Comparator sites were chosen because they had between 4000 and 10,000 total attendances in September 2021.

| **Accident and Emergency service provider** | **September 2021 attendance count** |
| --- | --- |
| James Paget University Hospitals NHS Foundation Trust | 7,135 |
| The Queen Elizabeth Hospital, King's Lynn, NHS FT | 6,487 |
| Whittington Health NHS Trust | 9,275 |
| Birmingham Women's And Children's NHS Foundation Trust | 5,582 |
| Chesterfield Royal Hospital NHS Foundation Trust | 8,323 |
| George Eliot Hospital NHS Trust | 8,100 |
| South Warwickshire NHS Foundation Trust | 7,035 |
| Wye Valley NHS Trust | 5,676 |
| Airedale NHS Foundation Trust | 6,316 |
| Barnsley Hospital NHS Foundation Trust | 8,872 |
| Gateshead Health NHS Foundation Trust | 8,355 |
| Harrogate And District NHS Foundation Trust | 5,586 |
| Sheffield Children's NHS Foundation Trust | 5,490 |
| Alder Hey Children's NHS Foundation Trust | 5,910 |
| Countess Of Chester Hospital NHS Foundation Trust | 7,353 |
| East Cheshire NHS Trust | 4,453 |
| Mid Cheshire Hospitals NHS Foundation Trust | 8,981 |
| Isle Of Wight NHS Trust | 5,371 |
| Royal Surrey County Hospital NHS Foundation Trust | 6,928 |
| Dorset County Hospital NHS Foundation Trust | 6,302 |
| North Bristol NHS Trust | 8,392 |
| Royal United Hospitals Bath NHS Foundation Trust | 7,706 |
| Salisbury NHS Foundation Trust | 6,172 |
| Torbay And South Devon NHS Foundation Trust | 8,731 |
| Yeovil District Hospital NHS Foundation Trust | 5,296 |

**List S2.**

**Patient Satisfaction Questions (invited to contribute after service completed)**

# GP Front Door - Patient feedback (existing service monitoring)

You have been seen today in a Primary Care service. This is a separate service to Accident and Emergency (A&E)

We would like to ask you a few short questions regarding your experience today. Should you wish to feedback further on your experiences, you will have to opportunity to leave your contact details if you wish at the end.

1.Who referred you to the Emergency Department today

You came yourself (self-referral)

Your GP advised you to attend

You called 111 and they advised you to attend

Other (details requested)

2.Were you aware of this trial Service before you arrived today?

Yes

No

Other (details requested)

3.How did you find the person you met in the Emergency Department (our Streamer)

Excellent

Good

Fair

Poor

Very Poor

Other (details requested)

4. How helpful did you find our Receptionist who booked you in?

Very helpful

Somewhat helpful

Neither helpful nor unhelpful

Somewhat unhelpful

Very unhelpful

Other (details requested)

5.How did you find the consultation with the Clinician / Doctor

Excellent

Good

Fair

Poor

Very Poor

Other (details requested)

6. Would you recommend our Service to your friends and family?

Yes

No

Other (details requested)

7. How do you think we should improve our Service? (Free text answer)

8. We are running an evaluation alongside this service for us to learn from it and hopefully improve the service further. Should you choose to provide your email and phone number and contact name below we may well contact you for further insight into your experience of the service that you have engaged with today.

Please add any further comments (Free text answer)

**Figure S1. Histograms showing frequency distributions of key parameters (JPUH and QEH patients combined)**

S2a. Wait time (arrival to being seen: in minutes, data shown were truncated at 100 minutes, 93% of attendances shown

S2b. Distance from registered home address to service: in miles, data shown were truncated at 30 miles, 86% of arrivals shown

S2c. Age of patient: years

S2d. Deprivation decile : 10 categories from most to least deprived, in national distribution these are equal size groups but in N&W are clustered in deciles 3-7

| 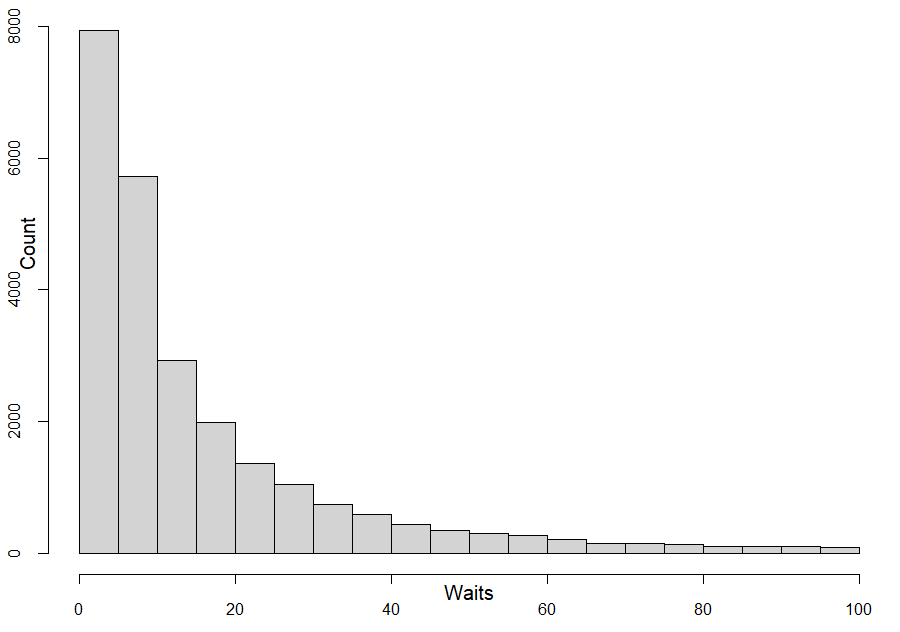  a. Wait time (arrival to being seen): 5 minute groups | 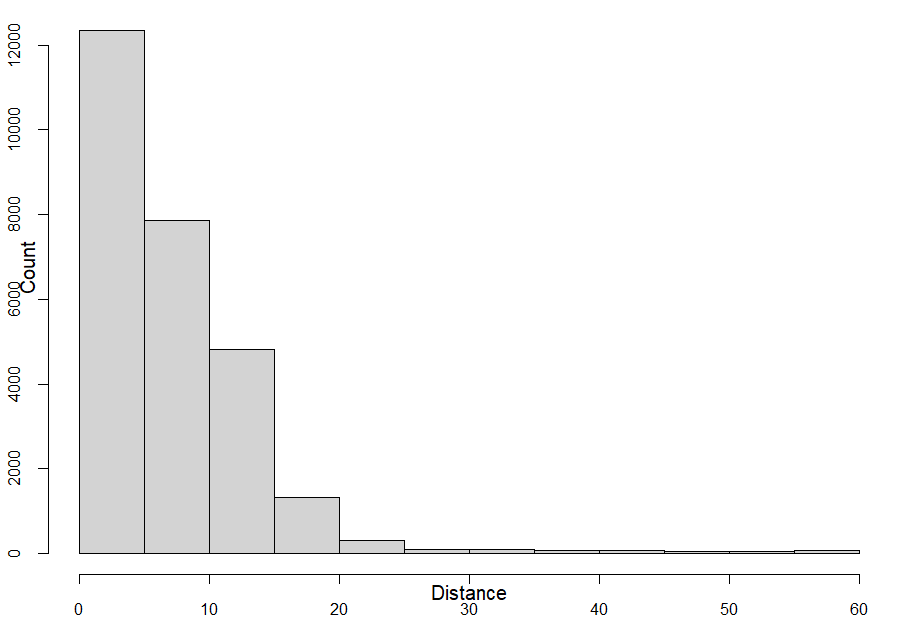  b. Distance from home address to service: 5 mile groups |
| --- | --- |
| 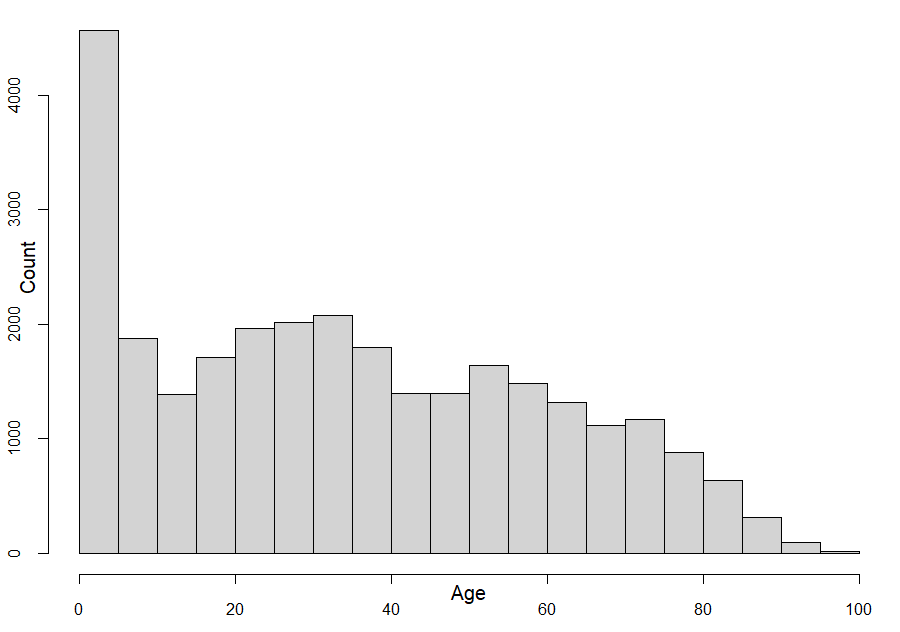  c. Age of patient: 5 year groups : 0-4, 5-9, 10-14 etc. | 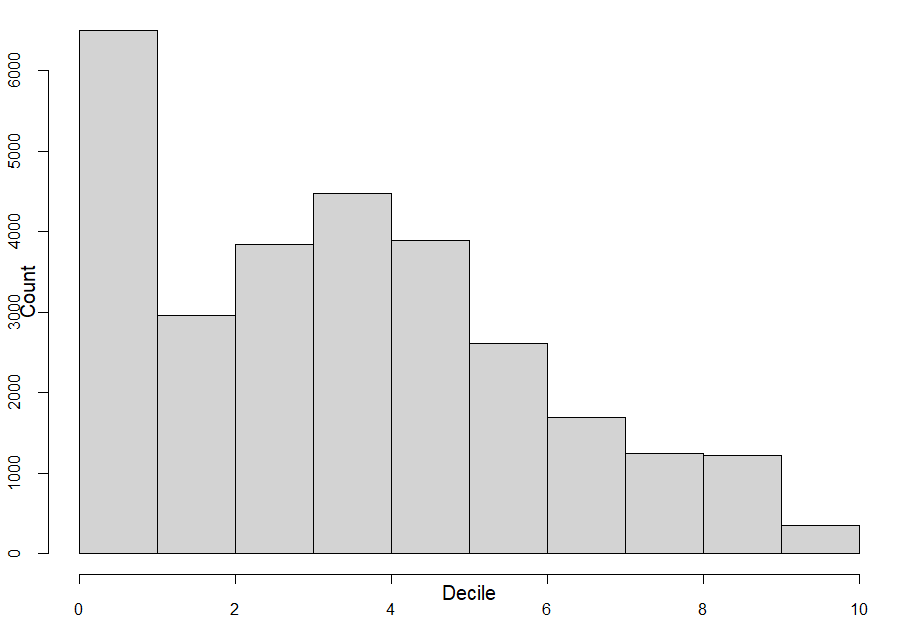  d. Deprivation decile |

**Figure S2. Attendance counts used in analysis of wait times** (data flow chart)

18,212 attendance records at JPUH +

10,655 attendance records at QEH = 28,867 total attendance records

One QEH attendance with no information about completion status and no wait time recorded

153 patients booked in who did not clearly complete their GDAE appointment but had indicative wait time information. Non completers were censored observations in survival analysis.

*Reasons for non completion of booked appointment*:

121 Patient did not attend including when patient cancelled or walked out

23 Cancelled by service

9 Booking made but not clear if attended

28,713 attendances that completed appointments with known outcomes : data used in Table 1 summary

28,866 attendances used to construct preferred model for wait times with (significant) co-variates in AFT regression : site and age

**Figure S3. Probability of having been seen after booking in (Kaplan Meier curve)**


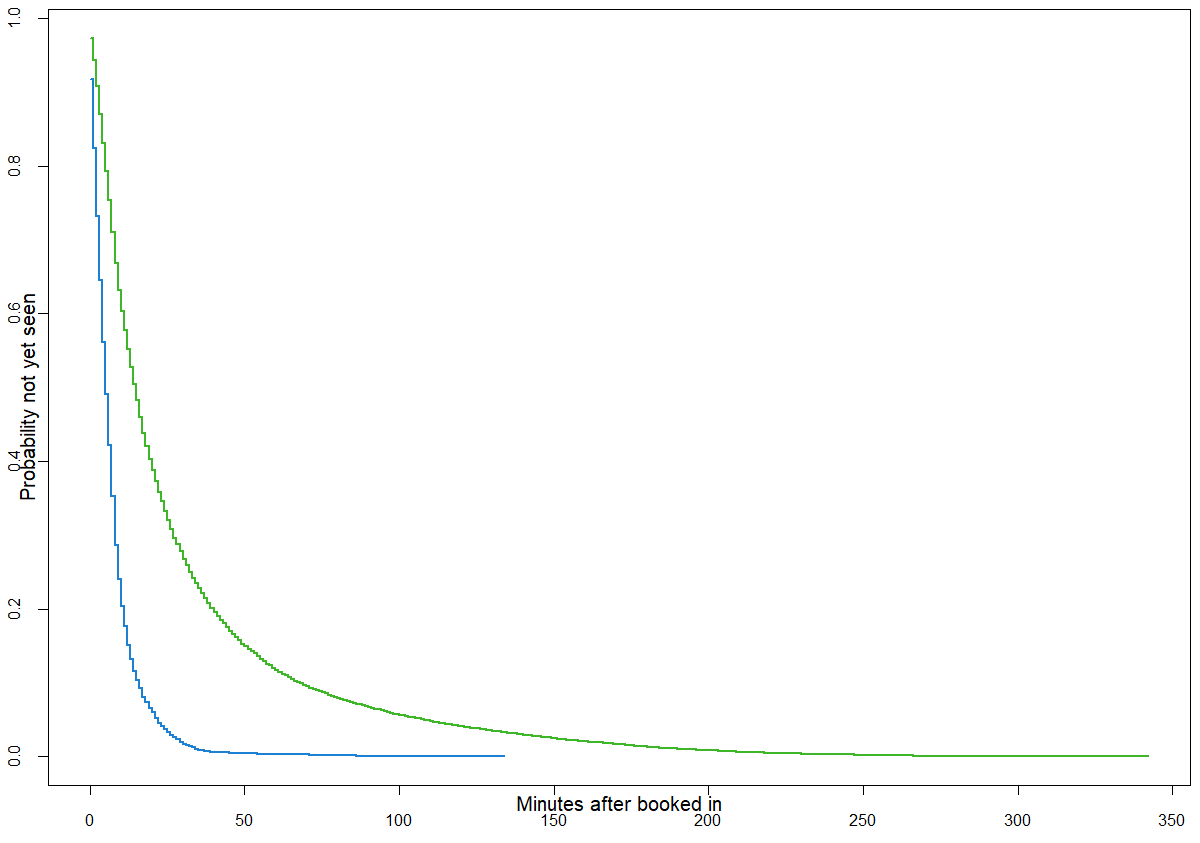


Key: Green: Queen Elizabeth Hospital; Blue: James Paget University Hospital

**Figure S4. Residuals for AFT model**


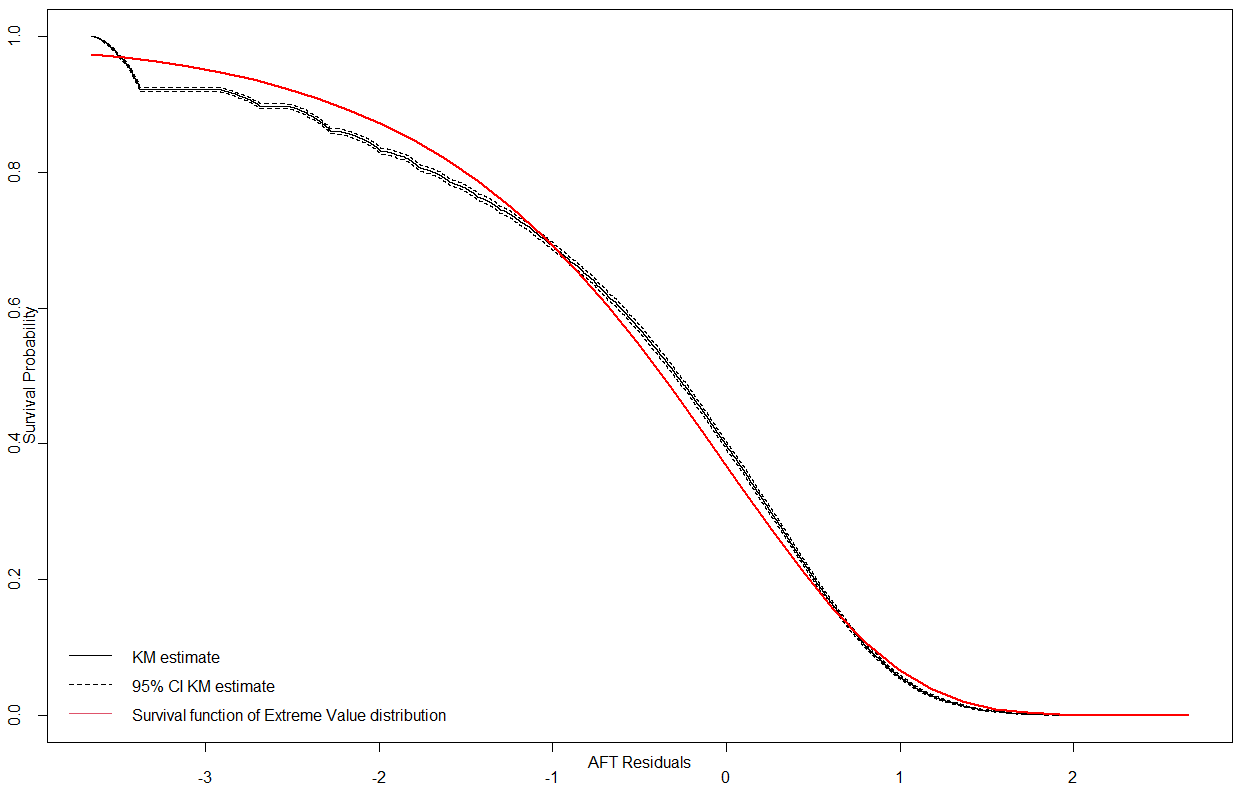


Note: KM = Kaplan Meier.
